# Trends in US Medicare Decedents’ Diagnosis of Dementia From 2004 to 2017

**DOI:** 10.1001/jamahealthforum.2022.0346

**Published:** 2022-04-01

**Authors:** Matthew A. Davis, Chiang-Hua Chang, Sharon Simonton, Julie P. W. Bynum

**Affiliations:** 1 Department of Systems, Populations and Leadership, University of Michigan School of Nursing, Ann Arbor; 2 Department of Learning Health Sciences, University of Michigan Medical School, Ann Arbor; 3 Institute for Healthcare Policy and Innovation, University of Michigan, Ann Arbor; 4 Department of Internal Medicine, University of Michigan Medical School, Ann Arbor

## Abstract

**Importance:**

Alzheimer disease and related dementias (ADRD) have received considerable attention among clinicians, researchers, and policy makers in recent years. Despite increased awareness, few studies have documented temporal changes in the documentation of ADRD diagnoses despite its new importance for risk adjustment for health plans in Medicare.

**Objective:**

To assess trends in frequency of ADRD diagnosis in the last 2 years of life from 2004 to 2017, as well as any associated changes in billing practices, characteristics of the population with diagnosed ADRD, and intensity of end-of-life care.

**Design, Setting, and Participants:**

This is a serial cross-sectional study of older adult decedents (67 years or older) from 2004 to 2017 using a 20% sample of fee-for-service Medicare decedents. An ADRD diagnosis within the last 2 years of life was identified using diagnosis codes from inpatient, professional service, home health, or hospice claims, requiring the standard claims algorithm that required at least 1 claim and a more stringent algorithm that required at least 2 claims. Trends in ADRD diagnosis among decedents were used to lessen influence of new diagnostic technologies for early stage disease. Demographic characteristics, selected comorbidities, place of death, and health service use at the end-of-life were also examined. Data were analyzed from July 9, 2020, to May 3, 2021.

**Exposures:**

Calendar year 2004 to 2017.

**Main Outcome and Measure:**

An ADRD diagnosis within 2 years of death.

**Results:**

Among the included 3 515 329 Medicare fee-for-service decedents, when adjusted for age and sex, the percentage of older decedents with an ADRD diagnosis increased from 34.7% in 2004 to 47.2% in 2017. The trend was attenuated (25.2% to 39.2%) using a stringent ADRD definition. There was an inflection in the curve from 2011 to 2013, the time at which additional diagnoses were added to Medicare claims and the National Alzheimer Care Act was enacted. The ADRD diagnosis frequency increased considerably in inpatient (49.0% to 67.3%), hospice (12.2% to 42.0%), and home health (10.1% to 28.7%) claims. However, individual characteristics, number of visits, and hospitalizations were similar across the study period, and the intensity of end-of-life care declined on most measures.

**Conclusions and Relevance:**

In this cross-sectional study, nearly half of older Medicare decedents had a diagnosis of ADRD at the time of death. From 2004 to 2017, the percentage of older adult decedents who received an ADRD diagnosis increased substantially prior to announcement of the addition of ADRD to Medicare risk adjustment strategies.

## Key Points

**Question:**

To what degree did the diagnosis of Alzheimer disease and related dementias (ADRD) change at the end of life between 2004 and 2017?

**Findings:**

Among 3 515 329 Medicare fee-for-service decedents, the percentage who received an ADRD diagnosis within 2 years of death increased from 34.7% in 2004 to 47.2% in 2017. The likelihood of receiving an ADRD diagnosis particularly increased in the inpatient, hospice, and home health settings; individual characteristics and service use were stable over time, while the intensity of end-of-life care declined on most measures.

**Meaning:**

Dying with an ADRD diagnosis has become more common among older US decedents, potentially owing to increased awareness and temporal changes in billing.

## Teaser

This cross-sectional study assesses trends in frequency of diagnosis of Alzheimer disease and related dementias in the last 2 years of life and any associated changes in billing practices, characteristics of the diagnosed population, and intensity of end-of-life care.

## Introduction

Alzheimer disease and related dementias (ADRD) for which we have no effective disease-modifying treatment are estimated to cost the US as much as $215 billion annually.^1^ As the population ages, the importance of ADRD as a cause of or contributor to death continues to increase.^2^ The recognition of dementia and the burden to affected individuals and their families has increased efforts to address the needs of this population. In addition to the human toll of the disease, there are also high costs associated with both medical care and long-term care as people with dementia progress from mild impairment toward death. The increased attention has led to reassessments of how to account for the presence of dementia in the processes leading up to death.

Recently, contributing factors such as ADRD have been taken into consideration when determining causes of death,^2,3^ and ADRD has increased from the eighth leading cause of death in the US in 2000 to the sixth in 2018^4,5^ (and the fifth among older adults^6^). This change reflects changing attitudes and practices for documenting the presence of this disease, which is also likely happening in clinical practice. Dementia has long been noted to be underdiagnosed,^7^ but higher levels of public education and more diagnostic and treatment options may be changing how often clinicians document and bill for ADRD. Moreover, several external factors could affect ADRD coding, such as the adoption of electronic health records, the influence of the National Alzheimer Project Act^8^ on disease awareness and increased research funding, and changes in the allowable number and coding systems used for diagnoses on Medicare claims.

Understanding whether ADRD identification has increased is important because it may suggest greater opportunity to address burdensome end-of-life care. But it also has health policy importance. Dementia was added into the risk adjustment strategy Hierarchical Condition Categories (HCC), constructed from algorithms of claims, and used by the Centers for Medicare & Medicaid Services for compensating health plans.^9,10^ In addition, ADRD coding is important for economic evaluations of how dementia contributes to the costs of care.

Therefore, the objective of this study was to determine whether the likelihood of dying with an ADRD diagnosis, identified from billing data, has changed over time. We examined diagnosis of ADRD among decedents because end-stage disease should be less influenced by temporal changes in diagnostic technologies used in early disease stages.^11,12^ The hypothesis was that ADRD diagnosis frequency would increase over time coincident with changes in diagnostic billing behavior. We examined whether the potential change in ADRD diagnosis frequency was associated with changes in the characteristics of the ADRD-identified population. Lastly, we examined whether trends toward greater diagnosis frequency were associated with changes in the intensity of care at the end of life.

## Methods

### Study Design

A serial cross-sectional study of Medicare decedents using a 20% national sample of fee-for-service enrollees from 2004 to 2017 was conducted to examine changes in the percentage of older adults receiving an ADRD diagnosis within 2 years of death. This study received approval from the institutional review board of Dartmouth College, waiving informed consent for the use of deidentified data, and abides by Strengthening the Reporting of Observational Studies in Epidemiology (STROBE) reporting guidelines.

### Study Population

Using the 20% Master Beneficiary Summary Files, we identified all individuals who died between 2004 and 2017. Among decedents, we restricted to those who were continually enrolled in both Medicare Parts A and B, and not enrolled in Medicare Advantage for 24 months before the date of death. With a 2-year look-back period for diagnosis identification, decedents must have been 67 years or older at the time of death to be included. We chose a 2-year look-back period because the majority of decedents having an ADRD diagnosis will receive at least 1 ADRD diagnosis on a claim within 2 years of death.^13^ The final sample consisted of 3 515 329 older decedents.

### Identification of Decedents With ADRD Diagnosis

The presence of an ADRD diagnosis was identified from inpatient records (Medicare Provider Analysis and Review [MedPAR]), professional service claims (carrier file and selected outpatient hospital claims for clinician visits in underserved settings), home health records, and hospice claims. An established list of *International Classification of Diseases, Ninth Revision, Clinical Modification* and *International Statistical Classification of Diseases and Related Health Problems, Tenth Revision *codes were used (eTable in the Supplement).

We applied a standard, previously validated claims algorithm that requires the presence of a single claim with an ADRD diagnosis^14,15^ rather than 2 claims, as used for other diseases, owing to concerns for underdiagnosis. We also applied a more stringent approach by requiring 2 or more claims with an ADRD diagnosis for professional services at least 7 days apart. The latter approach reduces potential misclassification by removal of those who receive a spurious ADRD clinical diagnosis as in the case of rule-out diagnoses.^16,17,18^

Changes in clinical practice or volume of care delivered in each setting could influence frequency of diagnosis documentation or setting where diagnosis occurred across the inpatient, professional service, home health, or hospice settings. In addition to identifying how many decedents had received an ADRD diagnosis, we examined whether changes had occurred in the setting where the diagnosis was billed. Trends were examined in the occurrence of an ADRD diagnosis in each claim type (ie, nonmutually exclusive), as well as when the diagnosis appeared in only 1 claim type. For descriptive purposes, we superimposed the external events (adoption of electronic health records,^19,20^ billing rule changes, and enactment of the National Alzheimer Project Act) that we hypothesized could have been associated with the likelihood of ADRD diagnosis.

### Demographic Characteristics

Changes in the likelihood of receiving an ADRD diagnosis could lead to differences in the composition of the ADRD decedent population. We examined demographic characteristics and selected comorbidities among ADRD decedents relative to changes in decedents who did not receive an ADRD diagnosis. We examined characteristics in 2004 vs 2017 and in 2011 when the expansion in the number of diagnoses on health care claims went into effect.^21^ Demographic characteristics at the time of death included age, sex, and race and ethnicity. Race and ethnicity was categorized as Black vs non-Black because the percentage of Black and non-Black individuals was stable over time, unlike categorization of White race, which declined as coding of Hispanic ethnicity increased.^22^ Non-Black race and ethnicity included American Indian or Alaska Native, Asian, Hispanic, White, and those of other races and ethnicities. The Elixhauser Comorbidity Index was used to identify clinically significant comorbidities in the last year of life,^23^ including cancer, diabetes, congestive heart failure, chronic pulmonary disease, and kidney failure.

### Volume of Health Service Use

We also determined whether health service use temporally increased because previous research shows that more services would lead to a higher likelihood of diagnosis.^24^ Number of outpatient services (evaluation and management visits,^25^ emergency department visits, and home health days) and inpatient service use (inpatient evaluation and management visits, intensive care unit [ICU] stays, inpatient days, and skilled nursing facility days) were assessed 1 year before death.

### End-of-Life Treatments

Based on the MedPAR file, we report place of death as occurred in a (1) hospital without an ICU stay, (2) hospital with an ICU stay, or (3) some other type of Medicare-reimbursed inpatient stay (primarily a skilled nursing facility). We classified all other decedents with no MedPAR services on the date of death as unknown owing to limits of Medicare data, but they were most likely to have died in a community setting or long-term care nursing home. Any claim for hospice services in the last 6 months of life was used to determine the percentage of decedents who used hospice. Lastly, life-prolonging treatment used in the last 6 months of life included gastrostomy feeding tubes, mechanical ventilation, and hemodialysis.^26^

### Statistical Analysis

Overall trends from 2004 to 2017 in the percentage of decedents who received an ADRD diagnosis were age- and sex-adjusted using direct rate adjustment (with 2011 as the reference year). We used univariate statistical tests to compare the characteristics of decedents with an ADRD diagnosis over time (2004 vs 2017) and with decedents who did not receive an ADRD diagnosis in 2017. Statistical significance for trends in ADRD diagnosis across time was assessed by linear regression using calendar year as a continuous variable. All analyses were completed using SAS, version 9.4 (SAS Institute Inc). We set the critical α level to a 2-sided .05. Owing to the large sample size, even small differences can reach statistical significance.

## Results

### Trends in ADRD Diagnosis Among Decedents

**Figure 1.**
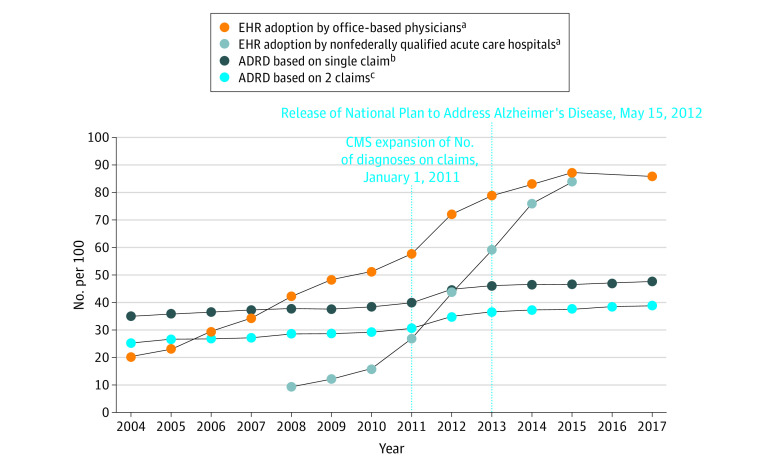
Age- and Sex-Adjusted Trends in the Percentage of Older Adult Medicare Decedents With an ADRD Diagnosis Within the Last 2 Years of Life, 2004-2017 ADRD indicates Alzheimer disease and related dementias; CMS, Centers for Medicare & Medicaid Services; EHR, electronic health record. ^a^Data on EHR adoption for office-based physicians and nonfederally qualified acute care hospitals are from the Office of the National Coordinator for Health Information Technology.^19,20^ ^b^ADRD identified based on a single claim within the last 2 years of life. ^c^ADRD identified based on 2 diagnoses on claims occurring more than 1 week apart within the last 2 years of life.

Among the 20% sample of Medicare fee-for-service decedents from 2004 to 2017, when adjusted for age and sex the percentage of decedents who received an ADRD diagnosis increased from 34.7% in 2004 to 47.2% in 2017, which equates to a 36.0% increase over the 14 years (*P* < .001; Figure 1^19,20^). Use of the more stringent definition of ADRD attenuated the estimates but not the percentage increase: the age- and sex-adjusted percentage of decedents who received an ADRD diagnosis increased from 25.2% to 39.2% in 2017 (*P* < .001) for a 55.6% increase over time. While overall trends increased, the largest increase occurred from 2011 to 2013, when the Centers for Medicare & Medicaid Services expanded diagnosis codes on the claims and the National Plan to Address Alzheimer’s Disease was enacted.

**Figure 2.**
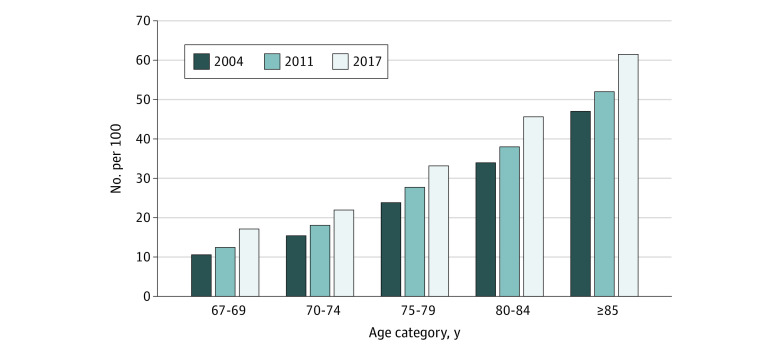
ADRD Diagnosis Among Older Adult Medicare Decedents by Age Category in 2004, 2011, and 2017 Alzheimer disease and related dementias (ADRD) were identified based on a single claim within the last 2 years of life.

By age category, decedents who were 85 years or older experienced the largest increase in ADRD diagnosis from 2004 to 2017. The percentage diagnosed with ADRD increased from 46.9% (55 074 of 117 409 decedents) in 2004 to 61.6% (71 162 of 115 605 decedents) in 2017 (Figure 2). Decedents aged 67 to 69 years also experienced a large 61.3% increase, albeit at lower absolute frequency, rising from 10.6% (1533 of 14 524 decedents) in 2004 to 17.1% (2429 of 14 180 decedents) in 2017.

### ADRD Diagnosis by Service Setting

**Figure 3.**
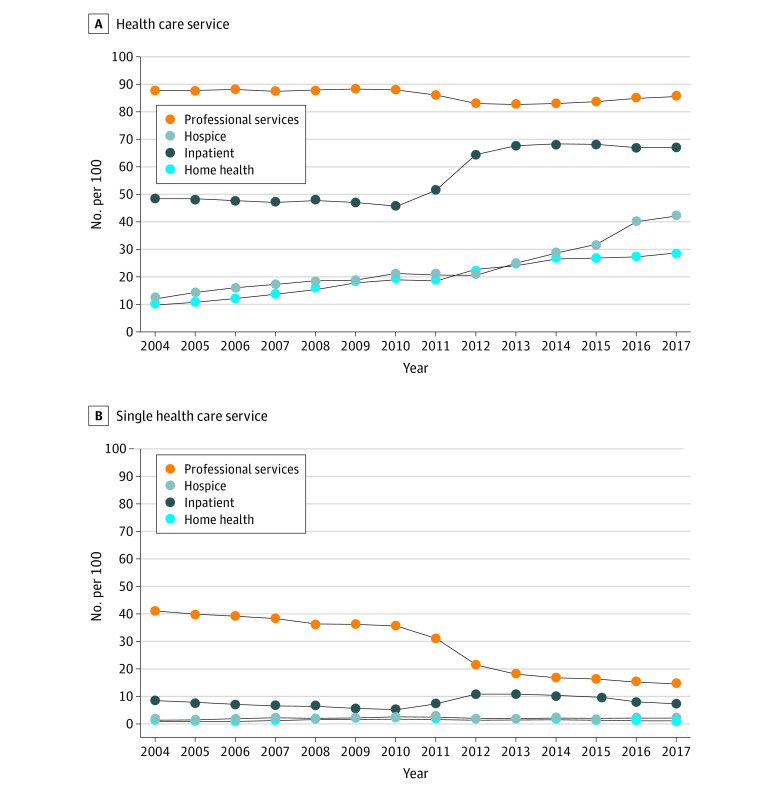
Trends in Older Adult Medicare Decedents Who Received an ADRD Diagnosis by Health Care Service and Single Health Care Service in the Last 2 Years of Life, 2004-2017 ADRD indicates Alzheimer disease and related dementias.

Trends in the service types for which an ADRD diagnosis was billed have changed since 2004 in 2 main ways. The likelihood of receiving an ADRD diagnosis has increased considerably among the inpatient, hospice, and home health settings (Figure 3A). The percentage who received an ADRD diagnosis from an inpatient service increased from 49.0% (45 759 of 93 386 decedents) in 2004 to 67.3% (74 277 of 110 368 decedents) in 2017 (*P* < .001), with all of the increase occurring between 2010 and 2013. Likewise, the percentage of ADRD decedents receiving a diagnosis from a hospice service increased from 12.2% (11 393 of 93 386 decedents) in 2004 to 42.0% (46 354 of 110 368 decedents) in 2017 (*P* < .001). The second change is that the likelihood of receiving an ADRD diagnosis from only one service type statistically significantly decreased (51.7% to 25.8%), driven by a drop in receiving only claims for professional services, from 40.6% (37 914 of 93 386 decedents) in 2004 to 15.3% (16 886 of 110 368 decedents) in 2017 (*P* < .001; Figure 3B).

### Characteristics and Service Use Trends Among Decedents

In comparison with decedents without ADRD, the changes in demographics and comorbidities over time for the decedents with ADRD were very similar (Table 1). The age, sex, and race and ethnicity changes were small among decedents with ADRD and paralleled the changes in the non-ADRD group. The percentage-point change in each comorbidity was the same for decedents with ADRD compared with decedents without ADRD, although the level of comorbidities differed between the groups in expected ways owing to the nature of ADRD.

**Table 1.** Characteristics of Medicare Decedents With ADRD Diagnosis vs Those Without a Diagnosis in 2004, 2011, and 2017

Characteristic	2004		2011		2017		*P* value^b^	
	ADRD diagnosis^a^	No ADRD diagnosis	ADRD diagnosis^a^	No ADRD diagnosis	ADRD diagnosis^a^	No ADRD diagnosis	2004 vs 2017	ADRD vs no ADRD in 2017
Sample size, No.	93 386	179 615	98 401	148 452	110 368	124 946	NA	NA
Sociodemographic characteristics								
Age, mean (SD), y	85.9 (7.3)	81.3 (8.0)	86.6 (7.3)	81.7 (8.4)	86.7 (7.8)	80.9 (8.6)	<.001	<.001
Age category, No. (%), y								
67-69	1533 (1.6)	12 991 (7.2)	1702 (1.7)	11 912 (8.0)	2429 (2.2)	11 751 (9.4)	<.001	<.001
70-74	5182 (5.5)	28 630 (15.9)	5158 (5.2)	23 256 (15.7)	6645 (6.0)	23 473 (18.8)		
75-79	11 351 (12.2)	36 226 (20.2)	9792 (10.0)	25 503 (17.2)	11 489 (10.4)	23 102 (18.5)		
80-85	20 246 (21.7)	39 433 (22.0)	18 346 (18.6)	29 634 (20.0)	18 643 (16.9)	22 177 (17.8)		
>85	55 074 (59.0)	62 335 (34.7)	63 403 (64.4)	58 147 (39.2)	71 162 (64.5)	44 443 (35.6)		
Sex, No. (%)								
Female	60 063 (64.3)	94 255 (52.5)	62 293 (63.3)	75 975 (51.2)	67 333 (61.0)	60 607 (48.5)	<.001	<.001
Male	33 323 (35.7)	85 360 (47.5)	36 108 (36.7)	72 477 (48.8)	43 035 (39.0)	64 339 (51.5)		
Race, No. (%)^c^								
Black	8655 (9.3)	14 433 (8.0)	8423 (8.6)	10 377 (7.0)	9115 (8.3)	8684 (7.0)	<.001	<.001
Non-Black	84 731 (90.7)	165 182 (92.0)	89 978 (91.4)	138 075 (93.0)	101 253 (91.7)	116 262 (93.0)		
Health conditions, No. (%)^d^								
Cancer	17 817 (19.1)	89 955 (50.1)	17 073 (17.4)	73 759 (49.7)	20 390 (18.5)	66 272 (53.0)	<.001	<.001
Diabetes	32 719 (35.0)	68 681 (38.2)	38 082 (38.7)	63 990 (43.1)	60 090 (54.4)	70 869 (56.7)	<.001	<.001
Congestive heart failure	41 083 (44.0)	81 672 (45.5)	37 722 (38.3)	59 640 (40.2)	43 063 (39.0)	50 475 (40.4)	<.001	<.001
Chronic pulmonary disease	29 643 (31.7)	71 858 (40.0)	29 820 (30.3)	57 225 (38.5)	32 617 (29.6)	47 053 (37.7)	<.001	<.001
Kidney failure	11 109 (11.9)	25 906 (14.4)	25 399 (25.8)	42 308 (28.5)	34 509 (31.3)	40 480 (32.4)	<.001	<.001
Health service associated with ADRD claim, %								
Any inpatient service	49.0	NA	51.8	NA	67.3	NA	NA	NA
Any professional service^e^	87.7	NA	86.3	NA	87.1	NA	NA	NA
Any hospice service	12.2	NA	21.5	NA	42.0	NA	NA	NA
Any home health service	10.1	NA	19.6	NA	28.7	NA	NA	NA
Only from inpatient service	8.4	NA	7.6	NA	7.5	NA	NA	NA
Only from professional service^e^	40.6	NA	31.3	NA	15.3	NA	NA	NA
Only from hospice service	1.5	NA	2.5	NA	2.2	NA	NA	NA
Only from home health service	1.2	NA	1.7	NA	0.8	NA	NA	NA

Abbreviations: ADRD, Alzheimer disease and related dementias; NA, not applicable.

^a^ Diagnosis of ADRD among decedents based on at least 1 ADRD claim within 2 years of death.

^b^ *t* Test was used for continuous measures and χ^2^ test for proportions.

^c^ Race and ethnicity was categorized as Black vs non-Black because the percentage of Black and non-Black individuals was stable over time. Non-Black race and ethnicity includes American Indian or Alaska Native, Asian, Hispanic, White, and those of other races and ethnicities.

^d^ Based on Elixhauser Comorbidity Index using diagnoses within 1 year of death.

^e^ Professional service identified using claims from outpatient and carrier files.

Between 2004 and 2017, there were changes in demographic characteristics within the ADRD decedent population worth noting (Table 1). While there were increases in both the oldest and youngest age, the average age of decedents with ADRD increased from 85.9 years in 2004 to 86.7 years in 2017 owing to a greater number of oldest old identified in claims. Other changes were consistent with the non-ADRD population change but are important to note for their potential influence on future ADRD studies that use longitudinal data. Among decedents with ADRD, the percentage of female decedents decreased from 64.3% in 2004 to 61.0% in 2017. The percentage of decedents with ADRD who were Black also decreased from 9.3% in 2004 to 8.3% in 2017. There were notable increases in the percentage of decedents with ADRD with diabetes (35.0% in 2004 vs 54.4% in 2017) and kidney failure (11.9% in 2004 vs 31.3% in 2017).

We found that the number and type of health service contacts increased for some types of care but not for the services from which most ADRD diagnosis claims are occurring. The number of outpatient evaluation and management visits, which are face-to-face visits with a clinician, increased slightly from 14.3 visits per decedent in the last year of life in 2004 to 15.9 visits in 2017 (*P* < .001; Table 2). The number of emergency department visits was low but increased from 0.8 per decedent in 2004 to 1.2 in 2017.

**Table 2.** Use of Health Care Services in the Last Year of Life Among Medicare Decedents With an ADRD Diagnosis in 2004, 2011, and 2017

Characteristic	Year			*P* value (2004 vs 2017)^a^
	2004	2011	2017	
Sample size with ADRD diagnosis, No.	93 386	98 401	110 368	NA
**Health service use in last year**				
Outpatient services, mean No.				
Outpatient EM visits	14.3	14.8	15.9	<.001
ED visits	0.8	1.0	1.2	<.001
Home health days	25.0	38.7	40.6	<.001
Inpatient services, mean No.				
Inpatient EM visits	12.1	11.4	10.9	<.001
ICU stays	3.2	3.8	3.8	<.001
Inpatient days	15.3	13.6	11.8	<.001
SNF days	21.6	27.2	22.9	<.001
**Intensity of end-of-life care**				
Place of death, %				
Hospital without ICU stay	12.3	7.8	5.4	<.001
Hospital with ICU stay	11.3	10.6	9.6	
Other inpatient	10.9	8.5	6.1	
Unknown	65.6	73.2	78.9	
Hospice use in last 6 mo, %				
Yes	36.4	54.6	62.7	<.001
No	63.6	45.4	37.3	
Life-prolonging treatment in last 6 mo, %				
On ventilator	6.8	7.2	7.6	<.001
With feeding tube	5.7	4.5	3.7	<.001
Received hemodialysis^b^	2.2	2.4	2.7	<.001

Abbreviations: ADRD, Alzheimer disease and related dementias; ED, emergency department; EM, evaluation and management; ICU, intensive care unit; SNF, skilled nursing facility.

^a^ *t* Test was used for continuous measures and χ^2^ test for proportions.

^b^ *International Statistical Classification of Diseases and Related Health Problems, Tenth Revision* codes for dialysis not used for 2016 Medicare Provider Analysis and Review data.

### Intensity of Care at the End of Life

From 2004 to 2017, there was a shift toward lower ICU use during a terminal hospitalization among decedents with ADRD (Table 2). Those who died in the hospital without an ICU stay also decreased from 12.3% in 2004 to 5.4% in 2017, whereas death in the community or other non-Medicare reimbursed setting (ie, the unknown category) increased from 65.6% to 78.9% during the same time period. Hospice use increased considerably from 36.4% in 2004 to 62.7% in 2017. Changes from 2004 to 2017 in life-prolonging treatment in the last 6 months of life were mixed: use of ventilation increased from 6.8% to 7.6%, whereas the use of feeding tubes decreased from 5.7% to 3.7%.

## Discussion

This study documents that ADRD diagnosis at the end of life is common and has increased from 2004 to 2017 among Medicare beneficiaries such that nearly half of fee-for-service Medicare beneficiaries die with a documented ADRD diagnosis. This is despite few changes in the characteristics of decedents with an ADRD diagnosis relative to the changes in the non-ADRD population, suggesting greater identification of previously undiagnosed cases. Most of the increase in ADRD diagnoses occurred in 2011 through 2013 when there was a change in the number of diagnoses allowed on Medicare claims. Of note, the transition from *International Classification of Diseases, Ninth Revision, Clinical Modification* to *International Statistical Classification of Diseases and Related Health Problems, Tenth Revision *diagnostic codes in 2015 was not associated with much change in frequency of ADRD diagnosis. There is some evidence that the settings in which ADRD was diagnosed have changed. In more recent years, decedents were more likely to receive an ADRD diagnosis from an inpatient, hospice, or home health service. As a corollary, we found a drop in diagnosis only in professional services claims, decreasing from 40.6% to 15.3%. Despite greater recognition of dementia in the last years of life, the use of life-prolonging services such as mechanical ventilation and dialysis have not declined even while more people with dementia are receiving hospice and fewer dying in hospitals or with feeding tubes.

To our knowledge, these analyses are among the first to examine trends in ADRD diagnosis among Medicare decedents. Prior cross-sectional studies estimate 40% of decedents with ADRD using Medicare claims,^13^ 37.7% with ADRD using claims and survey report,^27^ and 21.7% with dementia and 43.1% with cognitive impairment without dementia using cognitive testing.^28^ We find higher rates—47.2% of decedents with ADRD when using the standard single-claim algorithm and still 39.2% when we increase stringency by requiring 2 claims. The high and increasing rate of ADRD diagnosis, particularly for services often used in late-stage disease (ie, hospice, home health, hospital) and without the identified ADRD population characteristics changing differentially from the general population suggest that rates of underdiagnosis for late-stage disease may be declining. That said, identification within the last 2 years of life is a minor improvement considering the disease course for ADRD spans 10 years.^29^

One potential benefit of greater disease identification in late-stage disease is the opportunity to engage in end-of-life care discussions. The present results do not support an association between higher ADRD diagnosis frequency and less aggressive end-of-life care. Instead, we find that the secular trends identified by other studies are evident for the ADRD decedent populations, including the general population toward more hospice use,^30^ lower use of feeding tubes among non–community dwelling decedents with ADRD,^31^ and lower likelihood of dying in hospital albeit with high rates of between-facility transfers.^32,33,34^

From a health policy perspective, the implications of changes in ADRD diagnosis leading up to death extend beyond the implications related to quality of care. Diagnoses of ADRD were added to the HCC used for risk adjustment by the Centers for Medicare & Medicaid in 2020, though initially announced in December 2018.^35^ This study demonstrates that coding was increasing in fee-for-service (which is not subject to HCC adjustment) prior to this announcement. Further studies need to address the degree that these secular trends, along with new incentives to code ADRD by health plans, will have on costs to Medicare. Medicare claims have recently been shown to have a positive predictive value between 60% and 70%, which may make it challenging to disentangle upcoding, correction of historical underdiagnosis, and misdiagnosis.^36^ Additionally, 2 recent articles^36,37^ and the present analysis demonstrate that underdiagnosis may no longer warrant using 1 claim as opposed to the standard approach of using 2 in ADRD-diagnostic criteria. Thus, changes in ADRD diagnosis over time among Medicare decedents could affect payment and comparison of quality-of-care outcomes. Changes in the identification of ADRD at the end of life also likely affects both epidemiological measures of disease burden (eg, years of potential life lost) and estimates of the cost burden of ADRD to the US economy.

### Limitations

Several limitations must be acknowledged. First, while the present analyses demonstrate changes in ADRD diagnosis at the end of life among Medicare fee-for-service beneficiaries, these results may not extend to other groups (eg, beneficiaries enrolled in Medicare Advantage plans) or early stage disease. Second, inaccuracy of ADRD diagnoses in clinical practice and by extension in claims data likely leads to misclassification; nevertheless, these are the data used in most policy studies. Third, as an observational study we cannot rule out potential residual confounding affecting the results. Lastly, the analyses were not designed to determine whether the changes we observed are related to underlying population disease prevalence or lead to differences in clinical outcomes.

## Conclusions

In summary, in this serial cross-sectional study of Medicare decedents, the number of decedents dying with an ADRD diagnosis in the US increased by 36.0% between 2004 and 2017. Inpatient, hospice, and home health settings had the most statistically significant increases in ADRD diagnosis documentation. Individual characteristics and use of services, however, were relatively stable over time. These findings indicate that diagnosis of ADRD has become more common among older US decedents. Although these analyses are unable to fully explain such increases, it may be owing to increased awareness among patients, families, and/or health care professionals, as well as temporal changes in billing practices.

## Notes

Supplement. **eTable.** International Classification of Disease diagnosis codes used to identify ADRD
