# Supplementary material for: Trends in US Medicare Decedents’ Diagnosis of Dementia From 2004 to 2017
Source: JAMA Health Forum. 2022 Apr 1;3(4):e220346. doi: 10.1001/jamahealthforum.2022.0346 (PMC8976239; doi:10.1001/jamahealthforum.2022.0346)
Supplement: Supplement. — eTable. International Classification of Disease diagnosis codes used to identify ADRD [file jamahealthforum-e220346-s001.pdf]

Supplemental Online Content

Davis MA, Chang CH, Simonton S, Bynum JPW. Trends in US Medicare decedents' diagnosis of dementia from 2004 to 2017. *JAMA Health Forum*. 2022;3(4):e220346. doi:10.1001/jamahealthforum.2022.0346

eTable. International Classification of Disease diagnosis codes used to identify ADRD

This supplemental material has been provided by the authors to give readers additional information about their work.

© 2022 Davis MA et al. *JAMA Health Forum*.

eTable. International Classification of Disease diagnosis codes used to identify ADRD

ICD-9	Description	ICD-10	Description
290.0	Senile dements, uncomplicated	F015.0	Vascular dementia
2901	Presenile dementia	F01.51	Vascular dementia
290.10	Presenile uncomp	F02.80	Dementia in other diseases classified elsewhere
290.11	Presenile dementia with delirium	F02.81	Dementia in other diseases classified elsewhere
290.12	Presenile dementia with delusions	F03.90	Unspecified dementia
290.13	Presenile dementia with depression	F03.91	Unspecified dementia
290.2	Senile dementia with delusions or depression	F10.27	Alcohol dependence with alcohol induced persisting dementia
290.20	Senile dementia with delusions	G30.0	Alzheimer's disease
290.21	Senile dementia with depression	G30.1	Alzheimer's disease
290.3	Senile demetia with delirium	G30.8	Alzheimer's disease
290.4	Vascular dementia	G30.9	Alzheimer's disease
290.40	Vascular dementia uncomp	G31.01	Pick's disease
290.41	Vascular dementia with delirium	G31.09	Other frontotemporal dementia
290.42	Vascular dementia with delusions	G31.83	Dementia with Lewy Body
290.43	Vascular dementia with depression	F04	Amnestic disorder due to known physiological condition
291.2	Alcohol dependence with alcohol induced persisting dementia	F10.26	Alcohol dependence with alcohol induced persisting amnestic disorder
294.1	Dementia classified elsewhere (any specific etiology)	G311	Senile degeneration of brain, not elsewhere classified
294.10	Dementia classified elsewhere (any specific etiology) without behavioral disturbance	G312	Degeneration of nervous system due to alcohol
294.11	Dementia classified elsewhere (any specific etiology) with behavioral disturbance	R41.81	Age-related cognitive decline
294.2	Dementia unspecified		
294.20	Dementia, Unspecified w/o behavioral disturbance		
294.21	Dementia unspecified		
331.0	Alzheimer's disease		
331.11	Pick's Disease		
331.19	Frontal dementia		
331.82	Dementia with lewy body		
290.8	Other non specified senile psychoses		
294.0	Amnestic disorder classified elsewhere (Korsakoffs)		
331.2	Senile degen of brain		
331.7	Cerebral degen classified elsewhere (etoh, beriberi, stroke etc)		
797	Senility without mention of psychosis		
331.89	Cerebral ataxia		

Abbreviations: ADRD, Alzheimer’s disease and related dementias

© 2022 Davis MA et al. *JAMA Health Forum*.
